# Microbial Load and Antibiotic Resistance Patterns of *Escherichia coli* and *Enterococcus faecalis* Isolates from the Meat of Wild and Domestic Pigeons

**DOI:** 10.3390/foods8110536

**Published:** 2019-11-01

**Authors:** Jorge Cordero, Carlos Alonso-Calleja, Camino García-Fernández, Rosa Capita

**Affiliations:** 1 Department of Food Hygiene and Technology, Veterinary Faculty, University of León, E-24071 León, Spaincarlos.alonso.calleja@unileon.es (C.A.-C.);; 2 Institute of Food Science and Technology, University of León, E-24071 León, Spain

**Keywords:** pigeon meat, microbial load, antibiotic resistance, *Escherichia coli*, *Enterococcus faecalis*

## Abstract

An expansion in the consumption of pigeon meat has occurred in recent years. However, little is known about microbial load and antibiotic resistance of this foodstuff. The hygiene status and the antibiotic resistance patterns (disc diffusion; Clinical and Laboratory Standards Institute, CLSI) of *Escherichia coli* and *Enterococcus faecalis* isolates from wild and domestic pigeon carcasses were investigated. Average microbial loads (log_10_ cfu/cm^2^) ranged from 1.40 ± 1.17 (fecal coliforms) to 3.68 ± 1.40 (psychrotrophs). The highest (*p* < 0.05) microbial loads were observed in domestic pigeons. No substantial differences were found between isolates from domestic and wild pigeons with regard to the prevalence of antibiotic resistance. Of the *E. coli* strains, 20.00% were susceptible, 25.00% showed resistance or reduced susceptibility to one antimicrobial and 55.00% were multi-resistant. Among the *E. faecalis* isolates, 2.22% were susceptible and 97.78% were multi-resistant. The greatest prevalence of resistance or reduced susceptibility among *E. coli* was observed for amoxicillin-clavulanic acid (20.00% strains), ampicillin (26.67%), streptomycin (55.00%) and tobramycin (20.00%). The prevalence of resistance or reduced susceptibility among *E. faecalis* ranged from 31.11% (trimethoprim/sulfamethoxazole) to 97.78% (erythromycin). Meat from pigeons is a major reservoir of antibiotic-resistant bacteria. The need for the correct handling of this foodstuff in order to reduce risks to consumers is underlined.

## 1. Introduction

Each year, over two million wild pigeons are hunted in Spain for human consumption. This amounts to approximately 1256 tons of meat [1]. In addition, there are farms that breed pigeons and, in 2004, these produced approximately 57 tons of meat. Moreover, this activity is on an upward trend in Spain, as the number of such farms rose from 535 in 2007 to 1717 in 2019 [2].

The expansion in the production of pigeon meat that has occurred in recent years justifies an interest in its hygiene status. Aerobic plate counts (APCs), psychrotrophs, *Enterobacteriaceae*, fecal coliforms, enterococci, *Micrococcaceae*, lactic-acid bacteria (LAB) and *Brochothrix thermosphacta* have been used in the meat and poultry industries for assessing microbiological safety, processing hygiene, potential shelf-life and the keeping quality of products [3,4,5].

*Escherichia coli* and *Enterococcus* spp. are common bacteria in the intestinal tract of human beings and animals. Most strains of such bacteria are not pathogenic and are seen solely as indicators of fecal contamination. However, between 10% and 15% of *E. coli* strains are pathogenic and are able to cause a wide range of food-borne or water-borne illnesses [6,7]. In 2017, *E. coli* was responsible for 48 (0.9%) out of a total of 5079 outbreaks of food-borne diseases in the European Union [8]. On the other hand, enterococcal species have emerged as major causes of nosocomial- and community-acquired infections because of their capacity to acquire virulence traits [9]. Both *E. coli* and *Enterococcus* spp. have a capacity to acquire antibiotic resistance genes, based on the efficient horizontal transfer mechanisms these microorganisms have developed over the course of time. Hence, strains of these bacterial groups have been recognized as reservoirs of antimicrobial resistance genes. This is a worrying fact in the context of public health, since there is a high likelihood of the transfer of genes to pathogenic bacteria. On the other hand, this characteristic also allows these bacterial groups to be used as sentinels for resistance to antibiotics, and for tracking the evolution of antibiotic resistance in different environments [10].

Resistance to antibiotics has grown at an alarming rate in recent years, so as to become one of the greatest threats to public health and one of the main challenges for medicine in the twenty-first century [11]. Food-producing animals are a possible source of antibiotic-resistant bacteria that can be transferred to humans along the food chain [12]. Contacts with species which transfer multidrug-resistant bacteria to humans provide a biological mechanism for the increase noted in antibiotic resistance genes in human populations [13,14]. In this context, the main concerns about meat are the possibility of the bacteria surviving as a consequence of insufficient cooking and the potential for cross-contamination of other foods through inappropriate handling. In addition, wild animals are of importance for antibiotic resistance in two ways. Firstly, they can constitute a reservoir of antibiotic-resistant bacteria and resistance genes, with the potential for long-distance dissemination throughout the environment. Secondly, human activities can influence the gut microbiota of wild species, which could mirror the spectrum of antibiotic resistance in humans, as well as in the environment [9].

Monitoring resistance to antibiotics is essential in obtaining information about the size of this problem and the way it is trending, as well as in planning any control measures introduced and checking their effectiveness. However, little is known about resistance to antibiotics in strains from domestic and wild pigeons in Spain. The aim of this research was to learn and compare the microbiological quality of meat from domestic and wild pigeons in northwestern Spain. In addition, the patterns of resistance to antibiotics in strains of *E. coli* and *Enterococcus faecalis* isolated from such products were determined.

## 2. Materials and Methods

### 2.1. Samples

Forty pigeon carcasses were analyzed. Of these, 12 samples were domestic pigeons (*Columba livia*) reared in captivity, and 28 were wild pigeons (*Columba palumbus*) that had been hunted. Wild birds were obtained directly from hunters. All of the samples came from six localities in the Provinces of León and Zamora in northwestern Spain. Immediately after death, the birds were plucked and eviscerated in a closed room under aseptic conditions. None of the pigeon samples had the intestine perforated. The carcasses were transported individually in an ice chest to the laboratory, where they were kept under refrigeration (4 °C). From death to analysis, a maximum of 24 h elapsed.

### 2.2. Microbiological Determinations

The breasts of the carcasses were used as sampling points, and only regions which were not wounded by lead shot were used. Each sample was prepared by excising a piece of skin 10 cm^2^ in area with a sterile knife blade and a template. The samples were placed in a sterile stomacher bag containing 90 mL of sterile 0.1% (*w/v*) peptone water (Oxoid Ltd., Hampshire, UK) and homogenized (Masticator IUL, Barcelona, Spain) for two minutes. Decimal dilutions in sterile 0.1% (*w/v*) peptone water were prepared from this homogenate. Table 1 shows the culture media, incubation conditions and references used for each of the microbial groups studied. All inoculations were carried out in duplicate. Plates with between 25 and 250 colonies (spread plate technique) or between 30 and 300 colonies (pour plate technique) were counted, and average counts were calculated.

### 2.3. Isolation and Identification of Escherichia coli and Enterococcus spp. Strains

A total of three to five typical colonies in violet red bile agar (VRBA) and in kanamycin aesculin azide (KAA) agar were taken for later identification. Strains were streaked onto plates of tryptone soy agar (TSA, Oxoid, Hampshire, UK) and then incubated for 24 h at 44 °C (strains from VRBA) or 42 °C (strains from KAA) in order to obtain pure cultures. Presumed *E. coli* were examined for colony and cell morphology, for Gram staining, and for oxidase and catalase activities. Strains corresponding to Gram-negative, catalase-positive and oxidase-negative bacilli were identified with the aid of a miniaturized *E. coli* test system (Liofilchem s.r.l., Teramo, Italy) in accordance with the manufacturer’s instructions. A total of 60 *E. coli* strains (32 from domestic pigeons and 28 from wild) were selected for further antibiotyping.

Presumed enterococci were identified on the basis of Gram staining, catalase production, growth at 10 °C and at 45 °C, and growth in the presence of 6.5% NaCl. Gram-positive catalase-negative cocci able to grow under these conditions were inoculated into microtubes of API 20 Strep (bioMérieux, Marcy L’Étoile, France) in accordance with the manufacturer’s instructions. Data interpretation was carried out using the Analytical Profile Index (API) database (V7.0) with apiweb™ identification software (bioMérieux, Marcy L’Étoile, France). A total of 45 *E. faecalis* strains, 16 from domestic pigeons and 29 from wild, were selected for further antibiotyping. All strains were stored at −50 °C in tryptone soy broth (TSB) with 20% glycerol.

### 2.4. Antibiotic Susceptibility Determination

The strains were investigated to determine their susceptibility to antibiotics on Muller–Hinton agar (MH, Oxoid, Hampshire, UK) using the disc diffusion method. The susceptibility of *E. coli* strains was tested against a panel of 16 antimicrobials of clinical importance: amoxicillin-clavulanic acid (AMC; 30 µg), ampicillin (AMP; 10 µg), ceftazidime (CAZ; 30 µg), cefotaxime (CTX; 30 µg), cefoxitin (FOX; 30 μg), imipenem (IPM; 10 µg), aztreonam (ATM; 30 µg), nalidixic acid (NA; 30 mg), ciprofloxacin (CIP; 5 µg), chloramphenicol (C; 30 µg), gentamicin (CN; 10 µg), amikacin (AK; 30 μg), streptomycin (STR; 10 µg), tobramycin (TOB; 10 µg), tetracycline (TE; 30 µg) and trimethoprim/sulfamethoxazole (SXT; 25 µg). The *E. faecalis* strains were investigated to determine their susceptibility to 14 antibiotics: ampicillin (AMP; 10 µg), ciprofloxacin (CIP; 5 µg), chloramphenicol (C; 30 µg), gentamicin (CN; 10 µg), kanamycin (K, 120 µg), streptomycin (STR; 10 µg), erythromycin (E; 15 µg), fosfomycin (FOS; 50 µg), nitrofurantoin (F; 300 µg), rifampicin (RD; 5 µg), teicoplanin (TEC, 30 µg), vancomycin (VA, 30 µg), tetracycline (TE; 30 µg), and trimethoprim/sulfamethoxazole (SXT; 25 µg). All the discs used were purchased from Oxoid (Hampshire, UK).

After incubation at 37 °C for 18 to 24 h, inhibition halos were measured and scored as susceptible, intermediate (reduced susceptibility) or resistant according to the Clinical and Laboratory Standards Institute (CLSI) [19] guidelines. *Escherichia coli* ATCC 25922 and *Staphylococcus aureus* ATCC 29213 were used as reference strains for antibiotic disc control.

### 2.5. Statistical Analysis

Microbial counts were transformed to log_10_ cfu/cm^2^. Data were compared by analysis of variance (ANOVA) techniques, using Duncan’s multiple range test to separate averages. The prevalence of resistant strains in wild and domestic birds was compared using Fisher’s Exact Test. Significance was determined at the 5% (*p* < 0.05) level. All the tests were carried out with the Statistica^®^ 8.0 package (Statsoft Ltd., Tulsa, OK, USA).

## 3. Results

### 3.1. Microbial Loads

Microbial counts obtained from pigeon meat can be seen in Table 2. Average counts (log_10_ cfu/cm^2^) varied between 1.40 ± 1.17 for fecal coliforms and 3.68 ± 1.40 for psychrotrophs. Figures for domestic pigeons ranged from 1.47 ± 2.17 log units for *B. thermosphacta* to 3.84 ± 2.04 log units for psychrotrophs. There would appear to be no studies relating to the meat of farmed pigeons that could be used in comparisons with these results. In earlier investigations of chicken carcasses and drumsticks obtained from slaughterhouses or retail outlets, counts higher than those in the present research were observed [4,5,20,21,22]. The low microbial counts found in meat from domestic pigeons as compared with chicken meat may be due to the fact that the pigeons studied were slaughtered individually, since they were killed at farms for own consumption, and were plucked, eviscerated and refrigerated immediately after death, with microbiological analyses performed within 24 h of slaughter. It has been demonstrated that microbial counts increase significantly as storage time lengthens [23].

Microbial counts obtained on meat from wild pigeons varied between 1.10 ± 1.11 log_10_ cfu/cm^2^ for fecal coliforms and 3.61 ± 1.04 log_10_ cfu/cm^2^ for psychrotrophs. These figures are similar to microbial counts reported by El-Ghareeb et al. [24] on the skin of the breast area of pigeons hunted in Egypt. The present results are also in agreement with values observed by El-Dengawy and Nassar [25] for wild quail carcasses in Egypt, and by Paulsen et al. [23] for hunted pheasants in the Slovak Republic. In contrast, higher microbial loads were observed by Mousa et al. [26] in frozen quail in Egypt. These authors reported values of 6.71 log_10_ cfu/g, for psychrotrophs, 3.93 log_10_ cfu/g for enterobacteria and 3.86 log_10_ cfu/g for coliforms. It should be noted, however, that comparations between reports should be considered with caution because the different ways to report microbial load (ufc/cm^2^ or ufc/g).

It must be pointed out that the evisceration and refrigeration of the carcasses of the wild pigeons tested were performed immediately after they were hunted. This may have had an influence over the low counts found. In a number of studies, the evisceration of game has often been carried out no earlier than several days after death. This raises concerns from the viewpoint of food hygiene, especially when the intestines have been perforated and fecal material released into the body cavity [27]. Moreover, microbiological analyses were carried out within a period not exceeding 24 h at most after the animal was shot, so that it was to be expected that no substantial microbial growth would have occurred.

The lowest levels (*p* < 0.05) for APC, *Enterobacteriaceae*, fecal coliforms, *Micrococcaceae* and LAB were observed in wild pigeons. The microbiological condition of the carcasses of hunted animals depends on several factors. Among other considerations, the health of the animal, the time before chilling, and the conditions under which carcasses are eviscerated, transported, stored, and processed have a critical influence over carcass hygiene. Moreover, small game animals such as pigeons are usually hunted with shotguns, ensuring death from multiple lead shots hitting the body, occasionally penetrating deeper tissues and intestines [27,28]. The low levels of microorganisms recorded in the meat of hunted pigeons in the present study indicate that the product had been correctly handled. Moreover, the findings suggest that wild pigeons had less superficial contamination than domestic pigeons that had been reared on farms with a high density of animals. On these same lines, several authors have observed that the higher the stocking density, the higher the microbiological counts found in poultry meat. This occurs mainly because close proximity in livestock farms facilitates surface contamination with fecal material [29].

Psychrotrophs are the microorganisms of choice for assessing the microbiological quality of refrigerated meat. Higher average figures were observed for psychrotrophs, incubated at 7 °C, than for APC, incubated at 30 °C. These findings coincide with those from earlier work relating to poultry and poultry preparations stored under refrigeration [21]. In the work being presented here, samples remained under refrigeration for approximately 24 h from death to analysis. The fact that higher levels of psychrotrophs were observed suggests that storage temperatures were low enough.

### 3.2. Antibiotic Susceptibility in E. coli Strains

It would appear that the present study was the first to demonstrate the presence of several antimicrobial resistances in *E. coli* and *E. faecalis* recovered from pigeon meat in northwestern Spain. A total of 60 *E. coli* isolates, 32 from domestic pigeon meat and 28 from wild pigeon meat, were screened for susceptibility to a panel of 16 antimicrobial substances of veterinary and human clinical significance. Twelve strains (20.00% of the total) were sensitive to all the antibiotics, 15 strains (25.00%) showed resistance or reduced susceptibility to one antibiotic, and 33 strains (55.00%) were multi-resistant (showing resistant or reduced susceptibility to two or more antibiotics). The presence of antibiotic-resistant *E. coli* strains in meat is a frequent finding [12].

The average number of resistances per *E. coli* strain was 0.57. If the strains with resistance and those with reduced susceptibility are grouped together, the number of resistances per strain was 2.03. The number of resistances was 0.19 for isolates from domestic pigeons and 1.00 for those from wild birds. If the grouping of resistance and reduced susceptibility mentioned above is applied, the figures are 1.53 resistances per strain in domestic birds and 2.61 in wild. The average number of resistances per *E. coli* strain observed in the present study was lower than the figures recorded by Logue et al. [30] in the United States. These researchers found that strains of enterobacteria originating in poultry meat were resistant to 4.0 antimicrobials on average.

Clinical treatment for infections is rendered complex when strains are resistant to antibiotics. Infections caused by multi-resistant bacteria are associated with high morbidity and mortality rates, together with an increase in the costs of treating infections [31,32]. Bacteria resistant to antibiotics can cause infections in humans through the consumption of foodstuffs when cross-contamination or incomplete cooking occur. Thus, the presence of multi-resistant bacteria in food is a cause for great concern [10].

**Figure 1:**
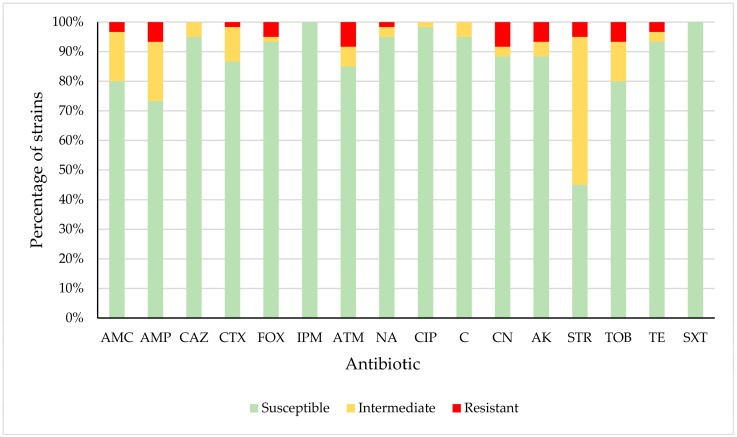
Percentage of *Escherichia coli* strains susceptible, intermediate or resistant to each antibiotic tested. Amoxicillin-clavulanic acid (AMC); ampicillin (AMP); ceftazidime (CAZ); cefotaxime (CTX); cefoxitin (FOX); imipenem (IPM); aztreonam (ATM); nalidixic acid (NA); ciprofloxacin (CIP); chloramphenicol (C); gentamicin (CN); amikacin (AK); streptomycin (STR); tobramycin (TOB); tetracycline (TE); trimethoprim-sulfamethoxazole (SXT).

Table 3 and Figure 1 show the percentages of *E. coli* strains that were susceptible, intermediate or resistant to each of the antibiotics used. All *E. coli* strains were susceptible to imipenem (IPM) and trimethoprim-sulfamethoxazole (SXT). Resistance was observed in *E. coli* strains relating to amoxicillin-clavulanic acid (AMC) (3.33% of strains), ampicillin (AMP) (6.67%), cefotaxime (CTX) (1.67%), cefoxitin (FOX) (5.00%), aztreonam (ATM) (8.33%), nalidixic acid (NA) (1.67%), gentamicin (CN) (8.33%), amikacin (AK) (6.67%), streptomycin (STR) (5.00%), tobramycin (TOB) (6.67%) and tetracycline (TE) (3.33%). Resistance or reduced susceptibility was observed in the case of AMC (20.00% of strains), AMP (26.67%), CAZ (5.00%), CTX (13.33%), FOX (6.67%), ATM (15.00%), NA (5.00%), CIP (1.67%), C (5.00%), CN (11.67%), AK (11.67%), STR (55.00%), TOB (20.00%) and TE (6.67%). High levels of resistance to such antimicrobials have also been reported in *E. coli* from domestic birds [33,34,35,36,37,38,39] and from various kinds of wild birds and mammals living in close proximity to humans, including pigeons [14,40,41,42,43,44,45,46,47,48,49,50,51,52,53].

It should be stressed that at least 20% of strains showed resistance or reduced susceptibility to amoxicillin-clavulanic acid, ampicillin, streptomycin and tobramycin, which are classified as “critically important antimicrobials for human medicine” [54]. According to the World Organization for Animal Health, these four antimicrobial compounds are categorized as “critically important veterinary antimicrobials” [55].

**Figure 2:**
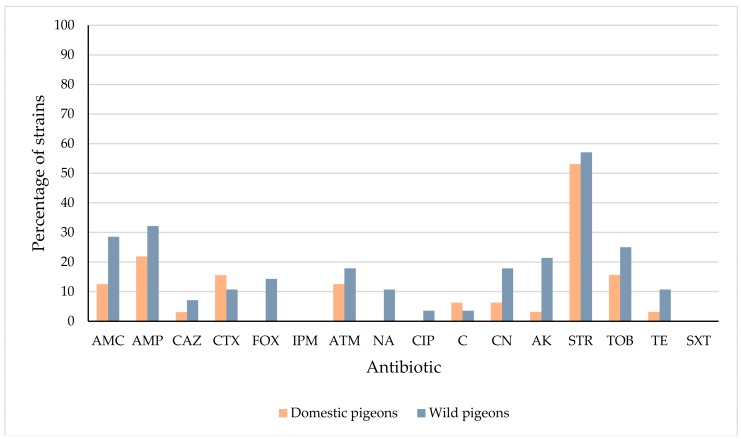
Percentage of *Escherichia coli* strains from domestic and wild pigeons with resistance or reduced susceptibility to each antibiotic tested. Amoxicillin-clavulanic acid (AMC); ampicillin (AMP); ceftazidime (CAZ); cefotaxime (CTX); cefoxitin (FOX); imipenem (IPM); aztreonam (ATM); nalidixic acid (NA); ciprofloxacin (CIP); chloramphenicol (C); gentamicin (CN); amikacin (AK); streptomycin (STR); tobramycin (TOB); tetracycline (TE); trimethoprim-sulfamethoxazole (SXT).

Data for resistance (including reduced susceptibility) in *E. coli* from domestic and wild pigeons are compared in Table 3 and Figure 2. Wild pigeons showed a higher (*p* < 0.05) percentage of resistant or intermediate strains than domestic pigeons in the case of FOX and AK. Similar (*p* > 0.05) percentages of resistant or intermediate *E. coli* strains were found in domestic and wild pigeons for all the remaining antibiotics. This is a surprising result, since it was to be expected that domestic birds would show higher levels of antibiotic resistance than wild pigeons. Contact with anthropogenic factors, such as human refuse or livestock farming, may encourage the colonization of birds by resistant bacteria, while also potentially allowing exposure to antimicrobial medication, antimicrobial residues, or resistant genes. This would contribute to the development and maintenance of antibiotic resistance in the microbiota of domestic animals [10,13,56,57]. The very slight differences noted here between domestic and wild pigeons in respect to resistance to antibiotics in isolates of *E. coli* underlines the impact of both livestock and human densities on the environment. It has been reported that a number of sites of human activities, such as human and veterinary clinical establishments, farms, landfills, or waste-water treatment facilities, may be the scene of interactions with wildlife, which may have a direct association with the antibiotic resistance profiles of bacteria of domestic and wild animals in any given geographic location [9]. It must be pointed out that the wild pigeons included in the present study came from zones close to human settlements and cattle farms, and so there would be a strong likelihood of contact and exchange of bacteria between pigeons, people and farm animals.

### 3.3. Antibiotic Susceptibility in E. faecalis Strains

Forty-five *E. faecalis* strains, 16 from domestic pigeons and 29 from wild, were screened for susceptibility to 14 antibiotics. Forty-four strains (97.78% of the total) were multi-resistant, having resistance or reduced susceptibility to two or more antibiotics, while just one strain (2.22%) was sensitive to all the antibiotics. The number of resistances per strain was 5.58. If strains with resistance and those with reduced susceptibility are taken together, the number of resistances per strain would rise to 10.49. The number of resistances per strain was similar in wild and in domestic pigeons. Thus, the figure for strains from domestic pigeons would be 5.38 if just resistance were to be considered, but 10.56 if both resistance and reduced susceptibility were considered together. For wild pigeons, the corresponding values would be 5.69 and 10.45, respectively. These data are similar to those previously recorded for Gram-positive bacteria in chicken meat, where an average of 6.35 antimicrobials was found for *Staphylococcus aureus* strains [5].

It should be noted that the average number of resistances per strain observed in the work being reported here was much higher for *E. faecalis* than for *E. coli* isolates. It has been suggested that *E. faecalis* has a striking ability to acquire and transfer antibiotic resistance genes [9]. Hence, *E. faecalis* is the enterococcal species that shows the highest average number of antimicrobial resistances per isolate [9,58,59,60].

**Figure 3:**
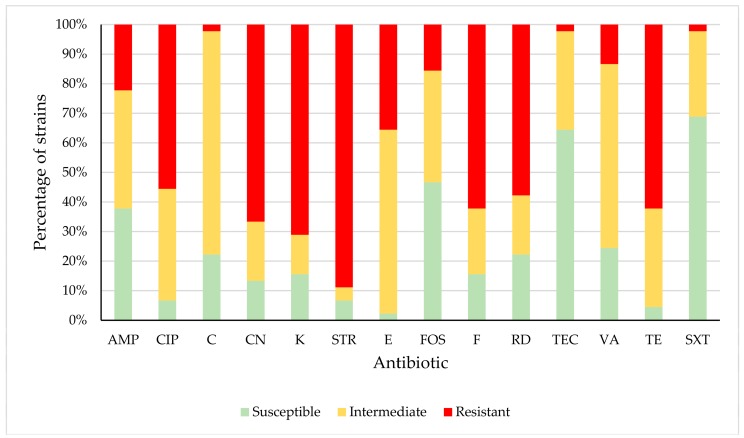
Percentage of *Enterococcus faecalis* strains susceptible, intermediate or resistant to each antibiotic tested. Ampicillin (AMP); ciprofloxacin (CIP); chloramphenicol (C); gentamicin (CN); kanamycin (K); streptomycin (STR); erythromycin (E); fosfomycin (FOS); nitrofurantoin (F); rifampicin (RD); teicoplanin (TEC); vancomycin (VA), tetracycline (TE); trimethoprim-sulfamethoxazole (SXT).

The considerable prevalence of resistant or intermediate *E. faecalis* strains observed in the work being reported here is worrying, since the resistances detected would probably undermine the usefulness as a therapeutic option of several antibiotics used in both human and veterinary medicine. *E. faecalis* isolates showed resistance to ampicillin (AMP (22.22% of strains), ciprofloxacin (CIP) (55.56%), chloramphenicol (C) (2.22%), gentamicin (CN) (66.67%), kanamycin (K) (71.11%), streptomycin (STR) (88.89%), erythromycin (E) (35.56%), fosfomycin (FOS) (15.56%), nitrofurantoin (F) (62.22%), rifampicin (RD) (57.78%), teicoplanin (TEC) (2.22%), vancomycin (VA) (13.33%), tetracycline (TE) (62.22%) and trimethoprim-sulfamethoxazole (SXT) (2.22%). Resistance or reduced susceptibility was observed for AMP (62.22% of strains), CIP (93.33%), C (77.78%), CN (86.67%), K (84.44%), STR (93.33%), E (97.78%), FOS (53.33%), F (84.44%), RD (77.78%), TEC (35.56%), VA (75.56%), TE (95.56%) and SXT (31.11%), as shown in Table 4 and Figure 3. These high percentages are of concern, because the above-mentioned antibiotics are categorized as “critically important antimicrobials” in the case of ampicillin, ciprofloxacin, gentamicin, kanamycin, streptomycin, erythromycin, fosfomycin, rifampicin, teicoplanin and vancomycin, as “highly important antimicrobials” in respect to chloramphenicol and tetracycline, or as “important antimicrobials” in the case of nitrofurantoin and trimethoprim-sulfamethoxazole, for human medicine [54]. Moreover, ampicillin, ciprofloxacin, gentamicin, kanamycin, streptomycin, fosfomycin, tetracycline and trimethoprim-sulfamethoxazole are classified as “veterinary critically important antimicrobials”, and rifampicin as a “veterinary highly important antimicrobial” [55]. High levels of resistance to such antimicrobials have also been reported in enterococcal strains from various domestic and wild animal species, including pigeons [9,14,45,46,51,61,62,63].

The great number of resistant strains in foods of animal origin mentioned in numerous publications would appear to be related to the use of antibiotics in animal production and clinical practice [10]. Thus, in the present study, a considerable prevalence of resistance was observed to antibiotics widely used in animal production [64,65,66]. It should be noted, however, that a high prevalence of resistance was also observed to substances whose use has been prohibited in food-producing animals for some decades, for instance, chloramphenicol or nitrofurantoin. Mechanisms of cross-resistance and co-resistance may have contributed to the persistence over time of genes for resistance to these substances, as previously suggested [10,35].

**Figure 4:**
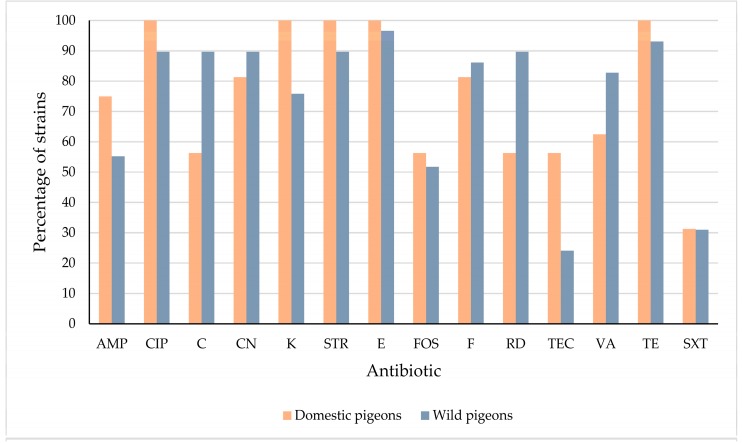
Percentage of *Enterococcus faecalis* strains from domestic and wild pigeons with resistance or reduced susceptibility to each antibiotic tested. Ampicillin (AMP); ciprofloxacin (CIP); chloramphenicol (C); gentamicin (CN); kanamycin (K); streptomycin (STR); erythromycin (E); fosfomycin (FOS); nitrofurantoin (F); rifampicin (RD); teicoplanin (TEC); vancomycin (VA), tetracycline (TE); trimethoprim-sulfamethoxazole (SXT).

Data for resistance or reduced susceptibility in *E. faecalis* from domestic and wild pigeons may be seen in Table 4 and Figure 4. Wild pigeons showed a higher (*p* < 0.05) percentage of resistant or intermediate strains than domestic pigeons in the case of C and RD. In contrast, the highest percentage of resistance or reduced susceptibility to TEC was observed for isolates from domestic pigeons. Similar (*p* > 0.05) percentages of resistant or intermediate *E. coli* strains were seen in strains from both domestic and wild pigeons in the case of all the other antibiotics.

## 4. Conclusions

Pigeon meat had low levels of all the microbial groups studied in comparison with the counts recorded in previous work with meat from other species of birds. The highest microbial loads were shown by pigeons reared in captivity, possibly as a consequence of high animal densities on farms. One worrying fact is the considerable prevalence of resistance to antibiotics observed among strains of *E. coli* and especially of *E. faecalis* isolated from pigeon meat. No substantial differences were noted between the levels of the prevalence of resistance to antibiotics in bacteria taken from the meat of domestic or of wild pigeons, which highlights the strong correlation between human activity and the spread of antibiotic resistance into wildlife.

The results from the present study provide evidence that bacteria from pigeon meat pose major potential risks, both direct and indirect, to consumers, in view of the considerable rates of resistance or of reduced susceptibility to antibiotics that were found. These results underline the importance of the careful handling of pigeon meat during preparation, avoiding cross-contamination and ensuring thorough cooking. The extensive prevalence of resistance to antibiotics found is a worrying fact from the viewpoint of food safety and public health, pointing to a need to take measures to reduce the rates of resistance to antibiotics among the bacteria present in pigeons.

## Figures and Tables

**Table 1:** Culture media, incubation times, temperatures and references used for microbiological analysis.

Microbial Group	Culture Medium	Incubation		Reference
		T (°C)	Time	
Aerobic plate count (APC) ^1^	Plate count agar (PCA)	30	72 h	[15]
Psychrotrophs ^1^	Plate count agar (PCA)	7	10 d	[16]
*Enterobacteriaceae* ^2,3^	Violet red bile glucose agar (VRBGA)	35	24 h	[17]
Fecal coliforms ^2,3^	Violet red bile agar (VRBA)	44	24 h	[17]
Enterococci ^2^	Kanamycin aesculin azide (KEA) agar	42	24 h	[17]
*Micrococcaceae* ^1^	Mannitol salt agar (MSA)	35	24–48 h	[18]
Lactic acid bacteria ^2^	De Man, Rogosa and Sharpe (MRS) agar	30	72 h	[17]
*Brochothrix thermosphacta* ^1^	Streptomycin thallous acetate actidione (STAA) agar	25	48 h	[18]

^1^ Spread plate technique (0.1 mL); ^2^ pour plate technique (1 mL); ^3^ overlay procedure. All media were purchased from Oxoid Ltd. (Hampshire, UK).

**Table 2:** Microbial counts (log_10_ cfu/cm^2^) on meat from domestic and wild pigeons in Spain.

	Type of Pigeon		
Microbial Group	Total	Domestic	Wild
Aerobic plate count (APC)	3.16 ± 0.95 _c_	3.76 ± 0.92 ^b^_b_	2.90 ± 0.84 ^a^_cd_
Psychrotrophs	3.68 ± 1.40 _d_	3.84 ± 2.04 ^a^_b_	3.61 ± 1.04 ^a^_e_
*Enterobacteriaceae*	1.56 ± 1.25 _a_	2.06 ± 1.19 ^b^_a_	1.35 ± 1.23 ^a^_ab_
Fecal coliforms	1.40 ± 1.17 _a_	2.11 ± 1.02 ^b^_a_	1.10 ± 1.11 ^a^_a_
Enterococci	1.82 ± 1.12 _a_	2.17 ± 0.85 ^a^_a_	1.67 ± 1.20 ^a^_b_
*Micrococcaceae*	2.73 ± 1.16 _b_	3.12 ± 1.05 ^b^_b_	2.56 ± 1.17 ^a^_c_
Lactic acid bacteria (LAB)	3.40 ± 1.04 _cd_	3.81 ± 0.93 ^b^_b_	3.23 ± 1.05 ^a^_de_
*Brochothrix thermosphacta*	1.53 ± 2.08 _a_	1.47 ± 2.17 ^a^_a_	1.56 ± 2.05 ^a^_ab_

Data (average ± STD) in the same row (superscripts) with no letters in common are significantly different (*p* < 0.05). Data in the same column (subscripts) with no letters in common are significantly different (*p* < 0.05).

**Table 3:** Number and percentage of *Escherichia coli* strains susceptible, intermediate or resistant to each antibiotic tested.

Antibiotic	Strains from Domestic Pigeons (*n* = 32)					Strains from Wild Pigeons (*n* = 28)					All Strains (*n* = 60)				
	Number of Isolates			% of Resistant Strains	% of Resistant and Intermediate Strains	Number of Isolates			% of Resistant Strains	% of Resistant and Intermediate Strains	Number of Isolates			% of Resistant Strains	% of Resistant and Intermediate Strains
	S	I	R			S	I	R			S	I	R		
AMC	28	4	0	0.00%	12.50%	20	6	2	7.14%	28.57%	48	10	2	3.33	20.00%
AMP	25	7	0	0.00%	21.88%	19	5	4	14.29%	32.14%	44	12	4	6.67	26.67%
CAZ	31	1	0	0.00%	3.13%	26	2	0	0.00%	7.14%	57	3	0	0.00	5.00%
CTX	27	4	1	3.13%	15.63%	25	3	0	0.00%	10.71%	52	7	1	1.67	13.33%
FOX	32	0	0	0.00%	0.00%	24	1	3	10.71%	14.29%	56	1	3	5.00	6.67%
IPM	32	0	0	0.00%	0.00%	28	0	0	0.00%	0.00%	60	0	0	0.00	0.00%
ATM	28	3	1	3.13%	12.50%	23	1	4	14.29%	17.86%	51	4	5	8.33	15.00%
NA	32	0	0	0.00%	0.00%	25	2	1	3.57%	10.71%	57	2	1	1.67	5.00%
CIP	32	0	0	0.00%	0.00%	27	1	0	0.00%	3.57%	59	1	0	0.00	1.67%
C	30	2	0	0.00%	6.25%	27	1	0	0.00%	3.57%	57	3	0	0.00	5.00%
CN	30	2	0	0.00%	6.25%	23	0	5	17.86%	17.86%	53	2	5	8.33	11.67%
AK	31	1	0	0.00%	3.13%	22	2	4	14.29%	21.43%	53	3	4	6.67	11.67%
STR	15	15	2	6.25%	53.13%	12	15	1	3.57%	57.14%	27	30	3	5.00	55.00%
TOB	27	4	1	3.13%	15.63%	21	4	3	10.71%	25.00%	48	8	4	6.67	20.00%
TE	31	0	1	3.13%	3.13%	25	2	1	3.57%	10.71%	56	2	2	3.33	6.67%
SXT	32	0	0	0.00%	0.00%	28	0	0	0.00%	0.00%	60	0	0	0.00	0.00%

Amoxicillin-clavulanic acid (AMC); ampicillin (AMP); ceftazidime (CAZ); cefotaxime (CTX); cefoxitin (FOX); imipenem (IPM); aztreonam (ATM); nalidixic acid (NA); ciprofloxacin (CIP); chloramphenicol (C); gentamicin (CN); amikacin (AK); streptomycin (STR); tobramycin (TOB); tetracycline (TE); trimethoprim-sulfamethoxazole (SXT).

**Table 4:** Number and percentage of *Enterococcus faecalis* strains susceptible, intermediate or resistant to each antibiotic tested.

Antibiotic	Strains from Domestic Pigeons (*n* = 16)					Strains from Wild Pigeons (*n* = 29)					All Strains (*n* = 45)				
	Number of Isolates			% of Resistant Strains	% of Resistant and Intermediate Strains	Number of Isolates			% of Resistant Strains	% of Resistant and Intermediate Strains	Number of Isolates			% of Resistant Strains	% of Resistant and Intermediate Strains
	S	I	R			S	I	R			S	I	R		
AMP	4	10	2	12,50%	75.00%	13	8	8	27.59%	55.17%	17	18	10	22.22%	62.22%
CIP	0	4	12	75.00%	100%	3	13	13	44.83%	89.66%	3	17	25	55.56%	93.33%
C	7	9	0	0.00%	56.25%	3	25	1	3.45%	89.66%	10	34	1	2.22%	77.78%
CN	3	6	7	43.75%	81.25%	3	3	23	79.31%	89.66%	6	9	30	66.67%	86.67%
K	0	2	14	87.50%	100%	7	4	18	62.07%	75.86%	7	6	32	71.11%	84.44%
STR	0	1	15	93.75%	100%	3	1	25	86.21%	89.66%	3	2	40	88.89%	93.33%
E	0	9	7	43.75%	100%	1	19	9	31.03%	96.55%	1	28	16	35.56%	97.78%
FOS	7	6	3	18.75%	56.25%	14	11	4	13.79%	51.72%	21	17	7	15.56%	53.33%
F	3	5	8	50.00%	81.25%	4	5	20	68.97%	86.21%	7	10	28	62.22%	84.44%
RD	7	3	6	37.50%	56.25%	3	6	20	68.97%	89.66%	10	9	26	57.78%	77.78%
TEC	7	9	0	0.00%	56.25%	22	6	1	3.45%	24.14%	29	15	1	2.22%	35.56%
VA	6	9	1	6.25%	62.50%	5	19	5	17.24%	82.76%	11	28	6	13.33%	75.56%
TE	0	5	11	68.75%	100%	2	10	17	58.62%	93.10%	2	15	28	62.22%	95.56%
SXT	11	5	0	0.00%	31.25%	20	8	1	3.45%	31.03%	31	13	1	2.22%	31.11%

Ampicillin (AMP); ciprofloxacin (CIP); chloramphenicol (C); gentamicin (CN); kanamycin (K); streptomycin (STR); erythromycin (E); fosfomycin (FOS); nitrofurantoin (F); rifampicin (RD); teicoplanin (TEC); vancomycin (VA), tetracycline (TE); trimethoprim-sulfamethoxazole (SXT).
